# Women's experiences and views about costs of seeking malaria chemoprevention and other antenatal services: a qualitative study from two districts in rural Tanzania

**DOI:** 10.1186/1475-2875-9-54

**Published:** 2010-02-17

**Authors:** Godfrey M Mubyazi, Paul Bloch, Pascal Magnussen, Øystein E Olsen, Jens Byskov, Kristian S Hansen, Ib C Bygbjerg

**Affiliations:** 1 National Institute for Medical Research (NIMR), Centre for Enhancement of Effective Malaria Interventions, Dar es Salaam, Tanzania; 2 Amani Medical Research Centre, Tanga, Tanzania; 3 DBL - Centre for Health Research and Development, Faculty of Life Sciences, University of Copenhagen, Denmark; 4 University of Aarhus, Department of Health Services Research, Institute of Public Health, Aarhus, Denmark; 5 University of Copenhagen, Department of International Health, Immunology and Microbiology, Denmark

## Abstract

**Background:**

The Tanzanian government recommends women who attend antenatal care (ANC) clinics to accept receiving intermittent preventive treatment against malaria during pregnancy (IPTp) and vouchers for insecticide-treated nets (ITNs) at subsidized prices. Little emphasis has been paid to investigate the ability of pregnant women to access and effectively utilize these services.

**Objectives:**

To describe the experience and perceptions of pregnant women about costs and cost barriers for accessing ANC services with emphasis on IPTp in rural Tanzania.

**Methods:**

Qualitative data were collected in the districts of Mufindi in Iringa Region and Mkuranga in Coast Region through 1) focus group discussions (FGDs) with pregnant women and mothers to infants and 2) exit-interviews with pregnant women identified at ANC clinics. Data were analyzed manually using qualitative content analysis methodology.

**Findings:**

FGD participants and interview respondents identified the following key limiting factors for women's use of ANC services: 1) costs in terms of money and time associated with accessing ANC clinics, 2) the presence of more or less official user-fees for some services within the ANC package, and 3) service providers' application of fines, penalties and blame when failing to adhere to service schedules. Interestingly, the time associated with travelling long distances to ANC clinics and ITN retailers and with waiting for services at clinic-level was a major factor of discouragement in the health seeking behaviour of pregnant women because it seriously affected their domestic responsibilities.

**Conclusion:**

A variety of resource-related factors were shown to affect the health seeking behaviour of pregnant women in rural Tanzania. Thus, accessibility to ANC services was hampered by direct and indirect costs, travel distances and waiting time. Strengthening of user-fee exemption practices and bringing services closer to the users, for example by promoting community-directed control of selected public health services, including IPTp, are urgently needed measures for increasing equity in health services in Tanzania.

## Background

Malaria chemoprevention during pregnancy or intermittent preventive treatment against malaria during pregnancy (IPTp) using sulphadoxine-pyrimethamine (SP) is implemented in Tanzania and other countries in Sub-Saharan Africa (SSA). A target was set by SSA governments through the Abuja Declaration of 2000 that by 2005 at least 60% of all pregnant women at risk of malaria should have access to IPTp services, and benefit from personal or community-protective measures such as insecticide-treated net (ITNs) [1]. Although actual coverage rates of IPTp and ITNs are debated there is a wealth of evidence indicating that pregnant women and their offspring have benefited tremendously from these interventions [2].

Early and continued attendance for ANC is greatly emphasized to enable the pregnant women receive the basic services including IPTp doses that to a great extent guarantee pregnancy safety, better health of the mother and improved delivery outcomes [3]. Sometimes new interventions are recommended while the existing policy framework or guidelines directly or indirectly limit the practicability of such interventions. For instance, in a situation whereby pregnant women book late or attend clinic irregularly due to perceived or real costs of seeking care, the timely delivery of IPTp doses becomes practically impossible. Therefore, it is imperative that as new intervention strategies are officially recommended analysis is done on the implications of the existing policy strategies on the feasibility and viability of such interventions in real world situation [4-6].

In 1993, the Government of Tanzania introduced a cost sharing policy for services delivered through public health facilities while emphasizing that basic maternal and child health (MCH) services should be delivered free of charge. Prior to this policy decision, that is, immediately after independence in 1961, the government had decided that the basic medical services at all the government/public facilities should be accessed without the service clients/users having to pay for them [7]. However, private health facilities continued imposing some fees for services until 1977 when the private medical practice was officially banned before its official re-institutionalization in 1991 [8]. Furthermore, in 2000 the government recommended that each pregnant woman attending MCH clinics should be given a voucher for procuring an ITN at a subsidized price. This step is line with the Abuja target of ensuring increasing coverage of pregnant women sleeping under ITNs [5]. Despite this equity-oriented political will renown as the government's long-term strategic principle of ensuring equity and universal access to primary health care [8,9], previous evaluations reports indicate that the free medical policy (user-fee exemption policy) is poorly adhered to due to lack of clear, feasible and/or disseminated guidelines [10-14].

The association between costs of accessing health services and low utilization by the poor is widely documented [2,15]. The poorest households/families in SSA face a greater cost burden due to malaria than wealthier ones, especially in rural areas [16-18]. Women living far away from public health facilities may be forced to access private health facilities including the private-for-profit facilities where services are commercialized and often charged at full price. The faith-based facilities also continued to operate privately but as they have not been profit motivated, they could offer services at subsidized prices and at times for free. However, the distribution of such facilities and the tendency of implementing user-fees or subsidies vary between regions [7,19]. Poor coverage of public health facilities in some, mainly rural, areas necessitates the presence of private sector providers in the delivery of health services [20]. Service providers in both the private-for-profit and private-not-for-profit (i.e. those owned by faith-based organizations (FBOs)) exit in Tanzania. As reflected in Table 1, public health facilities provide about 80% of all ANC services in Tanzania, the remaining services are provided by the private sector of which about 11% are covered by FBOs [21]. The nature of services provided at ANC visits are listed in Table 2.

**Table 1:** ANC services comprised within the National Guidelines on Focused ANC, Malaria and Syphilis in Pregnancy in Tanzania (Source: MOH 2004).

*Elements of Focused ANC package*	*Basic Practical Services*
**History Taking**: personal information, medical/surgical history, obstetrics and gynaecological history, ownership and use of an ITN **Physical Examination** General appearance, conjuctiva; weight; blood pressure; head to toe assessment including fundal height, lymph glands, gestational age, presentation, foetal lie, foetal heart sounds, genital exam; pelvic exam **Laboratory Tests** Urinary analysis (-albumen and sugar); Haemoglobin (Hb); blood grouping; rhesus factor; VDRL/RPR **Other general Services Information** Immunization and maternal medication information; delivery data, post-natal data, Sexually Transmitted Illnesses screening	- Early detection and management of disease/abnormality - Counselling on health promotion (including issues related to risks associated with poor nutrition, smoking, alcoholism, unsafe sex, hard work/lack of rest during pregnancy, lack of simple exercises, etc.), unauthorized use of drugs during pregnancy; lack of simple exercises, etc.); breastfeeding; personal hygiene - Administration of IPTp doses using SP (*taking history first about previous use of SP and side-effects, gestational age, educating the client about IPTp, administering IPTp to the eligible ones under direct observation*) - Birth preparedness and Individual Birth Plan -Danger signs and complication readiness i.e. *detection of danger signs and preparedness in case of complications* -Prevention of Mother To Child Transmission (PMTCT) of Human Immunodeficiency Virus (HIV) -Infection prevention i.e. *the measures to prevent possible infections that would affect the foetus and its mother* -Record keeping, referral and follow-up

**Table 2:** Distribution of health facilities and ownership in Tanzania

	Number by ownership					
***Type of Facility***	***Government***	***Parastatal***	***Voluntary***	***Private***	***Other***	***Total***

Specialized	4	0	2	0	0	6
Regional Hospital	17	0	0	0	0	17
District Hospital	55	2	13	0	0	70
Other Hospitals	2	6	56	20	2	86
Health Centres	49	6	48	16	0	479
Dispensaries	2,450	202	612	665	28	3,955
Specialized clinics	75	0	4	22	0	101
Nursing Homes	0	0	0	6	0	6
Private Laboratories	18	3	9	184	0	214
Private x-ray units	5	3	2	16	1	27

Total	3,035	22	746	927	31	4,961

Source: http://www.moh.go.tz/Health%20Indicators.php, as updated by the MOHSW on 7^th ^Dec 2009 and accessed on 8^th ^Dec 2009)

Costs of health services may be direct or indirect. Direct costs relate specifically to services such as diagnostics and treatment and the indirect costs may relate to transport, food and time in connection with health seeking actions [4,22,23]. However, little is known about cost barriers to IPTp delivery and uptake [24] and other ANC services in Tanzania [5] or elsewhere [2,4]. The present paper presents some of the findings from a larger study on the economic and other contextual determinants of acceptability and practicability of IPTp for malaria during pregnancy in Tanzania [5] augmenting the findings published recently based on interviews with national level officers [7]. ANC seeking behaviour of pregnant women living in rural district settings is described with emphasis on the influence of direct and indirect costs on accessibility to and utilizations of services, including IPTp.

## Methods

### Study design

This is a cross-sectional study on the perceptions and attitudes of pregnant women and mothers to infants on cost-related barriers to ANC services. Methods of data collection were qualitative and included Focus Group Discussions (FGDs) carried out at community level, semi-structured exit-interviews conducted at health facility level, and observations made within health facilities.

### Study areas

**Figure 1:**
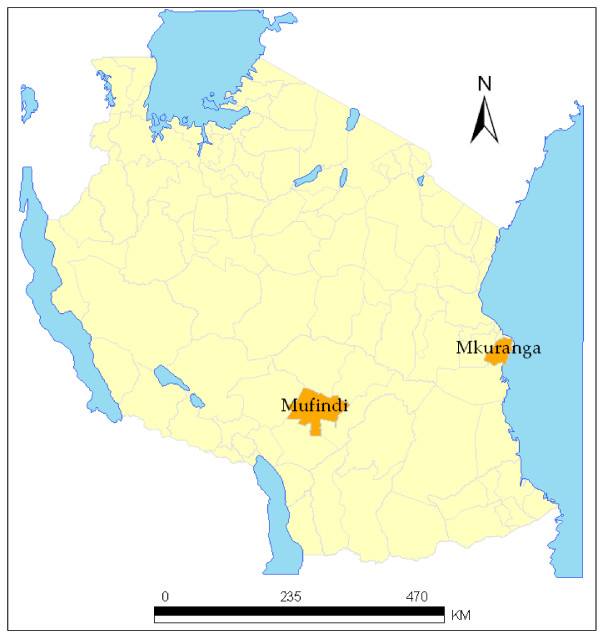
**Location of Mkuranga and Mufindi districts shown on the map of Tanzania**.

**Figure 2:**
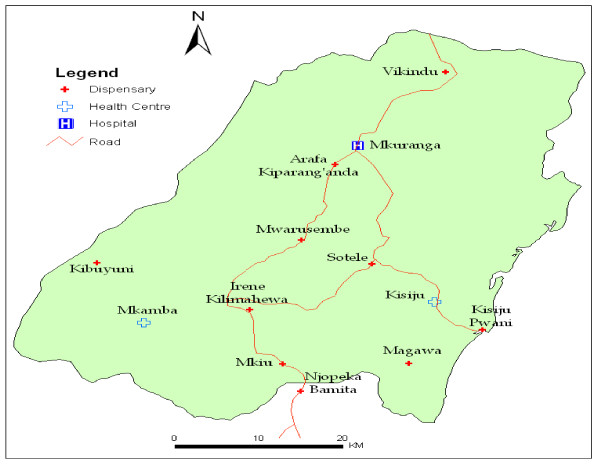
**Names and location of Health Facilities selected for study in Mkuranga district, Tanzania**.

**Figure 3:**
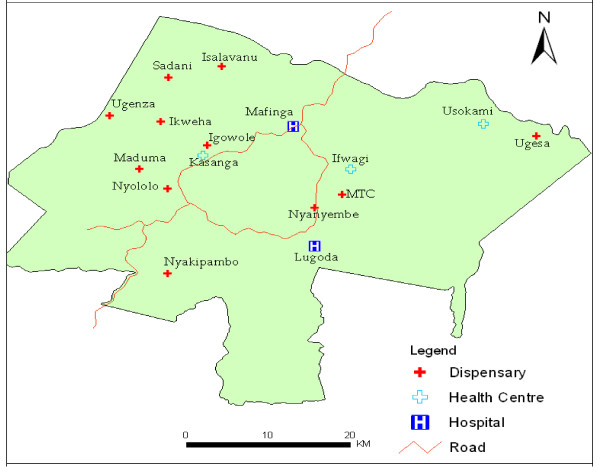
**Names and location of Health Facilities selected for study in Mufindi district, Tanzania**.

The study was undertaken between November 2005 and October 2006 in Mkuranga and Mufindi districts in Tanzania. Mkuranga district is located in the Coast Region, along the eastern coast of the Indian Ocean. It experiences stable and perennial malaria transmission throughout the year while Mufindi district is located in an area with unstable malaria transmission in the South-Western Highlands [25]. These districts were included due to their locations in different regions (Figure 1) with different malaria transmission intensities, socio-economic characteristics and health infrastructure profiles [26]. Both target districts are typically rural with poor health indicators and small-scale farming as the main occupation. At the time of the study, there were 54 health facilities in Mufindi. Five of these were private (either faith-based or commercial) health facilities delivering ANC services. In Mkuranga, there were 28 health facilities of which 6 were private with ANC clinics (Districts Annual Health Plans 2006, unpublished; per comm. with District Medical Officers (DMO)). Ethnically, Mkuranga is predominantly resided by the Zaramos, Ndegereko, Matumbi and Makonde tribes [27,28] whereas Mufindi is predominantly resided by the Hehe, Bena and Kinga tribes. Each of these tribes speaks different local languages and has local beliefs and traditional lifestyles that differ from those of other tribes. The locations of the health clinics/facilities visited in each district are shown (Figure 2 and Figure 3).

### Target groups and sampling approaches

At village level, the target groups were pregnant women and mothers with infants mobilized with help of local government leaders and village health workers (VHWs). These helpers were given specification beforehand regarding the characteristics of the participants targeted and how to select them within the village e.g. from different households randomly selected in each village regardless of income, ethnicity/tribe, education level and other too personalized characteristics. But in doing this, the helpers were accompanied by at least one member of the study team. It was assumed that village level participants comprised a mixture of women who used ANC services and women who did not. This was expected to stimulate discussion about barriers in health seeking behaviour. Six villages were randomly selected within each district, and within each village two groups, one with pregnant women and the other with mothers to infants, of 6-12 participants per group were assembled for FGDs. The total number of FGDs conducted in the study was thus 24. In Mkuranga, 48 pregnant women from six groups and 70 mothers of infant children from six groups participanted in the FGDs. In Mufindi, 50 pregnant women and 72 mothers of infant children participated in FGDs. The number of the participants per group ranged between 6 and 12 in all except in two groups (one in Mkuranga and one in Mufindi) whereby 14 mothers of infant children were involved per group. It was found socially appealing to involve a little more than 12 participants in these groups because turning back someone who has already volunteered sparing their time to attend a discussion could be psychosocially considered a humiliation or disrespect. The two groups were well managed even though a vast literature recommends that a FGD be limited to at-most 12 participants. Thus, the chosen number of study variables is a compromise between what is desirable and what is feasible [29].

Suspecting that asking the individual study participants to state their levels of incomes, education backgrounds and ethnicity would lower their confidence in contributing their ideas during the FGD session if they felt that the investigators were interested in individual rather than group's opinions, the demographic characteristics based on which the were mobilized were only limited to their pregnancy or motherhood status.

ANC clinics included in the study were both public (i.e. operated by government institutions) and private (i.e. operated by non-government institutions). Private clinics either operate for profit (i.e commercial facilities) or not for profit (e.g. faith-based clinics). Selection of ANC clinics followed a combination of randomization and convenient sampling techniques as described in the following:

In Mkuranga district there were only three health centres, one of which was owned by the Roman Catholic Church. This health centre was excluded from the sampling frame because it was new and thus underutilised (as reported by the officers in the DMO's office). The remaining two health centres were public and these were included in the study together with the single-standing public hospital available in the district. In addition, 10 dispensaries were randomly selected for the study. Five of these were private facilities (i.e. two not-for-profit-based and three profit-based). A total number of 417 pregnant women accepted to give (exit) interviews following their visit at an ANC clinic in Mkuranga district.

In Mufindi district the only two hospitals, one of which was public and urban, and the other private and rural (and non-profit-based), were included in the study together with a random selection of three health centres (two public and one private) out of a total of five health centres in the district (four of which were public). In addition, all 10 dispensaries providing ANC services in the district were included in the study. Seven of these dispensaries were public and three were private (two not-fo-profit-based and one owned by a private tea company providing health services to employees). All dispensaries were located in rural areas of the district. In total, 406 pregnant women accepted to give exit interviews in Mufindi district.

### Data collection techniques

FGDs were conducted at village level to obtain opinions about ANC clinics and services from pregnant women and from mothers to infants. These two groups were separated to minimize mismatch between current and previous experience. The FGDs were carried out using experienced moderators and observers. The discussions were tape-recorded and their transcription was coded thematically to allow a matrix sort of analytical framework to come up with the information that corroborated the hand-written notes taken during the discussions.

Exit interviews were carried out with pregnant women at ANC clinics immediately after receiving services. The interviews were based on semi-structured interview guides with a combination of open-ended and closed questions about direct and indirect costs of seeking ANC services.

### Data analysis

FGD and interview notes were checked for consistency and, when necessary, elaborated on the same day of collection. Both notes and tapes from FGDs were transcribed with assistance from an independent and experienced social scientist. The data were analyzed manually using qualitative content analysis and involved organizing the data in categories and themes for easy comparison and triangulation, as described elsewhere [7,29]. Several statements expressed by the study participants were quoted, some representing a common view/experience of several or all the members of the FGDs or an individual participant e.g. in the case of exit interviews.

### Ethical considerations

The district central and local government authorities expressed interest in the study and endorsed in writing that the study could be carried out in the selected districts. Ethical clearance was obtained from the National Medical Research Coordinating Committee under the Ministry of Health and Social Welfare (MoHSW) through Secretariat of the National Institute for Medical Research (NIMR). In line with national ethical guidelines [30], participants were provided with all relevant study information to assess their willingness to participate. This included study objectives, duration of participation, freedom to participate or withdraw from the study without being penalized or blamed, anonymity of participants, and plans for dissemination of the study findings. Consent to participate was provided orally.

## Results

From both FGDs and exit-interviews there was a relatively high level of satisfaction with the quality of ANC services. In particular, respondents acknowledged good interactions with most of the health workers as well as reasonably good physical conditions of clinic buildings, especially those operated by FBOs. However, mainly in the FGDs, a number of deficiencies with ANC services were highlighted. Some of these were related to costs and are addressed below.

### Accessing and costing transportation

From more than half of the women interviewed at clinic level and from the majority of the FGDs, concerns were raised about long travel distances to ANC clinics. This was considered a discouraging factor for regular ANC, and IPTp, service utilization.

"*We who come from Lugoda village usually spend four hours travelling before we arrive at the clinic because even if you have money there are no car" *(pregnant woman, Isalavanu public dispensary, Mufindi).

"*Today I have not been tested my blood at the clinic until next Wednesday*. *I am not sure if I will come back because I have other things to do at home and have come from very far*" (pregnant woman, Sotele public dispensary, Mkuranga).

Women living in rural areas faced greater difficulty with transport than women living in urban areas. In the rural areas of both districts, especially in rural Mkuranga, great concern were expressed with the shortage of public transport and this was reported to be contributed by poor road conditions that discourage private car owners to operate there. The situation gets more serious during rain season. In Mufindi, transport difficulties were mainly related to problems of payment (of bus fares or hiring bicycles) rather than availability of vehicles or poor road conditions. As confirmed by the district authorities and observed by the research team for the present study, the roads in Mufindi were generally good and passable throughout the year.

The out-of-pocket monetary costs to reach ANC clinics related to the payment of fares for buses, cars or motorcycles, or the hiring of bicycles. In both study districts, hiring of a bicycle cost the clients between 500 and 1000 shillings (US$1 ≅ US$ 0.83) per hour. Waiting time and delays at ANC clinics could thus have substantial direct costing implications for the renter, therefore, prompting some clients leave the clinic before receiving some important services, leave alone those who postponed attending clinic if they knew they could wait longer for services:

"*The government must consider people who come from villages that are very far way. Transport has cost me 1500 shillings today and still I have to pay for going back home*" (woman aged 38, Mafinga district hospital, Mufindi).

"*I would have gone to Mkuranga district hospital where better laboratory services and experienced staff are available, but I had no money for transport*" (woman aged 33, Arafa-Kiparang'anda private-for-profit dispensary, Mkuranga).

Procuring food or drinking water during transportation to and from ANC clinics was also reported being unavoidable and was considered an inconvenience by a number of the study participants. However, some of the participants argued that such costs were relatively small and would normally not deter women from attending services.

A special case on costs during transport comes from the small islands of Koma, Kwale, Dendeni and Duluti in the Indian Ocean where inhabitants must travel by boat for three or more hours to access the nearest ANC clinic of Kisiju-Pwani private-for-profit dispensary in Mkuranga district.

Waiting times may be extensive depending on weather conditions (waves and tides) and this may require ANC users to stay on the mainland overnight, often at a cost of accommodation and food.

If requiring laboratory testing or getting hold of a voucher for a subsidized ITN, the pregnant women would be referred to Kisiju-Kalole government health centre located at about 9.5 km away from the point of disembarkation. The transport fee by boat is 1000 shilling.

### Costs of referral

Health workers at dispensary level were accused of referring pregnant women to health centres or hospitals without any good reasons. This has substantial cost and time implications for the client who normally do not foresee or plan for such changes in the daily schedule. According to the respondents it was not uncommon that clients would be examined and discharged from the facility referred to with the message that nothing was wrong. Moreover, the required services at the facility referred to may, for various reasons, be missing.

"*Sometimes we are referred to Mafinga hospital for medical check-ups that could be managed here. I would advise that the staff at this clinic be confident about their skills because when we go to Mafinga we are disappointed to be told by the nurses there that our time and money have been wasted due to unnecessarily referrals*" (27-years old woman in her 3^rd ^pregnancy, Maduma public dispensary, Mufindi).

"*Here we don't get tested for the thing called Hb even if this is very important for a pregnant woman. We are referred to Mwalusembe Lutheran dispensary or Mkuranga district hospital or Kalole *[Kisiju public] *health centre both located very far away from us*" (38-years old woman in her 10^th ^pregnancy, Sotele public dispensary).

*"I have not had my blood tested because I was told that the responsible staff is absent. This is very bad because they should consider that we are tired of walking*" (20-years old pregnant woman, Sotele public dispensary, Mkuranga).

### User-fees at ANC clinics

Knowledge about the national cost-sharing policy and user-fee system in relation to ANC services varied among study participants in both districts. Some respondents informed that they had been paying for palpation, measuring body weight and blood pressure, ANC cards, SP for IPTp, and iron and mebendazole tablets at both public and private clinics. Whereas some respondents felt that they had to pay for almost all ANC related services, others admitted paying for some of the services, but not all.

"*We pay for every type of drug here, so the government has to look at this problem closely. Okay we are blamed for coming late to the clinic but what do they expect if we have no money?*" (woman aged 39, Nyololo public dispensary, Mufindi).

"*We usually do not pay for palpation, measuring blood pressure and weight, but do pay 200 shillings for laboratory test for urine, malaria, amount of blood, and ANC cards and this applies to government and private health facilities*" (FGD participants, Kitomondo village, Mkuranga)

The cost of an ANC card ranged from 200 to 500 shillings in Mkuranga and from 300 and 500 shillings in Mufindi. Alternatively the client would have to bring a notebook for keeping personal ANC records, and others were asked by the health workers to pay between 200-300 shillings for the notebook prepared for those who come without ANC cards at health clinic level.

At private profit-based clinics, payment for IPTp related services was almost always practiced. However, this was also commonly reported from public ANC clinics. In Mkuranga, pregnant women were commonly asked to bring their own drinking water for taking SP tablets for IPTp under direct observation by service providers. This was not a main problem in Mufindi where bottled water was occasionally supplied in support of IPTp services. It was commented:

"*They have just written for me to buy the drug and it is SP because I am sure of what I am saying and I know that SP is not panadol, paracetamol or any other drug*" (pregnant woman aged 20, Sotele public dispensary, Mkuranga).

Different opinions were expressed about user-fee implementation for ANC services at the study facilities. The presence of health workers who provided ANC services selectively by favouring some clients more than others was reported from both districts. As reported from Mkuranga, some people were totally denied ANC services in public facilities if they could not pay whereas others were given the services and allowed to pay later. To be denied services due to lack of money mainly frustrated the pregnant women who had come from far away and were late in their pregnancy. At the faith-based dispensary of Irene-Kilimahewa in Mkuranga district, it was official policy that HIV screening, costing between 1000 and 1200 shilling per person, could be paid later, if requested. This was reported to have a negative impact on early registration for ANC that lowers chances for the target women to receive the first dose of SP for IPTp.

**Figure 4:**
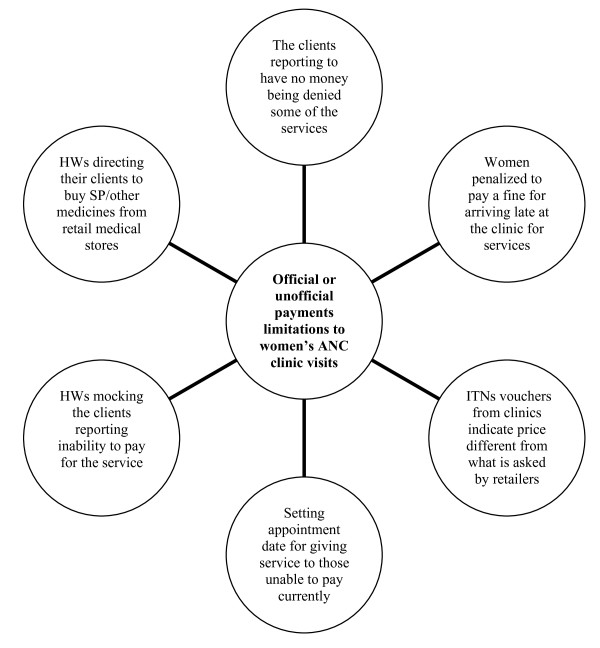
**Perceived barriers related to official and unofficial payments made by the women seeking for ANC services in Mkuranga and Mufindi districts, Tanzania**.

*"Nowadays government health facilities are like private ones because they charge for almost everything and the situation has worsened because it is very costly for a pregnant woman in a village like ours to attend clinic regularly until delivery*" (FGD with pregnant women, Mkerezange village, Mkuranga). Other disappointments are as summarized elsewhere (Figure 4).

"*Today I was asked to pay 1200 shillings but only had 700 shillings in my pocket. I paid what I had and got the service, but will pay the remaining amount when I come back to get the results next month*" (woman aged 26, Irene-Kilimahewa Catholic dispensary, Mkuranga).

### Unofficial payment and penalties

Various forms of unofficial and legally questionable service charges were reported to exist, more commonly at hospital and health centre level than at dispensary level. These charges were associated with the provision of acute services (e.g. when going into labour or requesting abortion), requests to procure drugs at private retailers based on arguments of stock depletion, or cash charges (of 100 shilling) to compensate village health workers for supporting outreach clinics.

*"We don't have the money for aborting our children as they request at... ...ldotsdispensary" *(woman in the mid-20's at one village level, Mkuranga), referring to staff at certain public clinics.

"*Our local leaders and health workers are carelessly setting fees that give them an opportunity to fill their stomachs at the expense of our sufferings. It is time for President Kikwete to visit us and listen to our cry*' (lactating mother at village level, Mufindi).

Moreover, pregnant women risked being penalized in terms of being fined or given some cleaning tasks if they did not adhere to their ANC schedules. At an FGD in Ihalimba village, Mufindi, it was complained that women who registered for ANC services late in their pregnancy risked receiving a fine of 1000 shilling. This discouraged women who, for any reason, had not registered in time to do so later in their pregnancy. Similarly, fines of 500 shillings for late registration were reported by FGD participants within other villages in Mufindi district. Moreover, the request for payment for services, whether official or unofficial, was humiliating for those pregnant women who were unaware about the costing and had, in an actual service situation, to declare their incapability to pay.

"*If you arrive late at the clinic, the staff rebukes and punishes you with a fine or they order you to clean the floor or sweep the surroundings" *(pregnant women, Nyololo village, Mufindi).

"*Sometimes when you tell the nurses that you have no money for the service they mock you and ask what you expect?" *(pregnant woman aged 22, at a private non-profit dispensary, Mkuranga).

### Waiting time and wasting time

Although acknowledging the deficiencies in human and material resources at most ANC clinics, hours of waiting for ANC services was considered a major problem affecting costs, satisfaction and perceptions of quality of health services. Moreover, pregnant women coming from far away found it particularly inconvenient and occasionally problematic when clinics opened later or closed earlier than officially announced. At times, clients missed ANC services due to such early closures of the clinics.

"*We pregnant women are complaining because they leave us helplessly at the clinic when they break for tea while we also have not taken anything by then*" (pregnant women, Mwalusembe village, Mkamba division, Mkuranga).

"*We plead the nurses to stop going to their houses and leaving us unattended for a long time*" (pregnant woman, Ugesa government dispensary, Kibengu division, Mufindi).

"*It is necessary for pregnant women to bring or buy something to eat since by nature of their conditions they should not stay for long without eating something*" (a respondent, Kisiju-Kalole public health centre, Mkuranga).

"*Seeing that they have already waited for a long time and still cannot get the service, some of the clients get disappointed and exchange bad words with the nurses. Shamelessly, the nurses become more furious and dare to frighten their clients for example by claiming..."Ok, wait you will come next time and find me here*" (a lactating mother during a FGD, confirmed by other participants, Mwalusembe Village, Mkuranga), referring to some staff normally at public clinics and at one faith-based clinic.

The combination of long travel distances and waiting times at clinic level made some respondents postpone their time of registration at ANC clinics.

"*If you book early, they tell you to come back every month. This will make you blame yourself for the time you waste and the tiredness you get out of travelling*' (woman aged 36, booked in the 28^th ^week of pregnancy, Arafa-Kiparang'anda private-for-profit dispensary, Mkuranga).

In contrast, efficiency in service delivery was a highly appreciated feature of ANC services.

"*The doctor *[in reality a clinical officer] *at our government hospital *[in reality a dispensary] *here at Isalavanu is a nice man*. *We don't know what will happen if he is transferred since when he is there, the services are delivered quickly and all the nurses behave well, but on the days he is absent the story of patients coming back at late ours repeats itself*" (lactating mother in her mid 30's, Lugoda village, Mufindi).

### Accessibility of subsidized ITNs

Respondents in both districts commended the government for introducing the national ITN voucher system. However, concerns were expressed about the need to approach accredited retailers, often located far away, for redeeming the ITN vouchers. Discrepancy between the actual price of the ITN and the discount indicated on the voucher was another source of concern.

In some villages (e.g. Nyololo, Maduma and Igowole in Mufindi district and Kitomondo, Mkerezange, Mwalusembe and Kiparang'anda villages in Mkuranga district) it was common to hire a bicycle to get hold of the ITN at the retailer. In Mufindi, it was noted that it could easily cost about 2000 shillings in transport to get hold of an ITN. If the ITNs or the vouchers were out of stock the cost (including the price of the net and indirect cost e.g. travel cost of accessing it) would be much higher:

"*I came for the first time and was told that the ITN vouchers were out of stock, the story was the same when I came for the second, third and fourth times and today it is my fifth time. I am tired of coming*" (woman aged 20, Mkamba public health centre, Mkuranga).

Psychologically, it would be humiliating to report lack of money for redeeming the ITN voucher:

"*If the nurses note that you are still having the voucher provided during the last visit they shout at you, so at times we wait until we get cash for the net before coming back to the clinic*" (a respondent aged 23, at one public dispensary, Mkuranga).

Nevertheless, positive remarks about the national ITN scheme were made by the respondents in both districts. As acknowledged by several study participants during FGD at village levels in both districts, the national ITN scheme had encouraged a considerable number of pregnant women to attend ANC clinics. The participants reported to have acquired the knowledge about this scheme and use of ITNs through health education at the ANC clinics and through radio announcements, billboards and other media sources.

## Discussion

The findings from the present study verify that ANC services in both public and private health facilities are far from being cost-free and this is contrary to the policy. Most respondents found themselves in a situation where they had to pay out-of-pocket, directly or indirectly, for accessing services. Even if there were no out-of-pocket costs associated with ANC services whatsoever, there would still be substantial opportunity costs and lost earnings associated with the spending of a considerable amount of time to access services [18,31]. This includes time lost on travel to and from the clinic and waiting time at the clinic.

Long travel and waiting time for service at the clinic appears to be a major concern to pregnant women and a key factor influencing decision on (i) whether to seek ANC services or stay at home, (ii) whether to register for ANC services early or late in pregnancy, and (iii) they type of health facility/provider to approach. On this basis it is not surprising if the rate of complete adherence to ANC schedules, corresponding to a minimum of four visits during the course of pregnancy [32,33], is poor. It is important and urgent to address the problems of long travel and waiting times from political level. This include a strong political will translated into action especially in terms of additional resource allocation geared for increasing the distribution of health facilities and human resources. Also, involvement of communities in service delivery, under the concept of Community-Directed Interventions (CDI) would be urgently useful. The CDI framework and approach has been successful in relation to various types of health services in several countries in SSA [34] and should, where possible, be considered for ANC services, including IPTp. A study carried out in Mukono district in Uganda documented that community health service volunteers were efficient in distributing SP for IPTp, providing health education, and referring pregnant women to formal clinics [35].

In Tanzania, it is still the government policy that all MCH including ANC services, except some of those requiring laboratory testing, are delivered free of charge in public health facilities [6].

Nevertheless, this study indicates that user-fees hamper the utilization of ANC services. Although there were different perceptions of which services were priced and which were free, and whether payment for services was official or unofficial and thus illegal, it was clear that the need to pay for services negatively affected the ANC seeking behaviour of pregnant women with implications for the regularity and timeliness of service provision, including IPTp. Similar observations were reported from studies carried out in Kenya and Malawi [36] as elsewhere in Tanzania [21,37,38]. Efforts to optimize IPTp services would therefore have to be coordinated with efforts to optimize ANC services at large.

The indication that unofficial payments may be requested for ANC services in Tanzania has also been observed in Kenya and elsewhere [4,39]. Although the magnitude of this problem is difficult to judge from the present study, there is a need for wide dissemination to health service providers and users of the rules and regulations for service fees, and the legal implications of violating them. This may also be seen as a concrete step towards enhancing transparency, accountability and public trust in the health system, as well as a measure to prevent unnecessary conflicts and increase compliance. Finally, improved communication with the health service user groups would increase public knowledge about the national cost-sharing policy including the rights of vulnerable groups to claim exemptions [10,11].

The application of fines, penalties, blame and rebuke by service providers that were reported to occur when the clients failed to adhere to their ANC schedules is professionally unethical, socially unacceptable, and must be addressed by health authorities. Better ways of promoting adherence to ANC services are suggested elsewhere in this paper.

The co-administration of IPTp and ITN interventions through ANC services may be conducive for maximizing the benefits of services to protect the pregnant women and their offspring. However, this study demonstrates the possible fragility of co-administration of interventions. In the event of any serious barrier to ANC service utilization either the provision of IPTp services or the acquisition of an ITN voucher, or both, may be missed. As an example, this study showed that failure to redeem and ITN voucher for a net could easily lead to postponement (if not termination) of future ANC clinic visits in fear of confronting rude staff. Fear of SP side effects in connection with the provision of IPTp services could lead to similar postponement or cancellation with serious implications on adherence to other ANC services including ITN acquisition. As pointed out by others, specific ANC services may not only negatively but also positively stimulate each other [2,40,41].

How a person believes or perceive of things in terms of health may influence his/her behaviour [26]. Thus, even though some of the issues discussed or perceived by the respondents/participants in the present study may be amplified or exaggerated contrary to the real situation, experts have argued that the client is the best judge of the service given rather than the service provider. In the present study, the issue of waiting time at the clinic was related to opportunity cost on the part of the client. If analysed from another viewpoint, it is also related to perceived quality of care. Whether it is the issue of quality of care or cost or both, the important point to bear in mind is that it is the clients who should be most respected of their views since they are the ones who are targeted for the services [42-46].

Furthermore, the present study verifies observation by other authors that use of qualitative approach in assessing poverty aspects related to population's health is useful to come up with the information that probably would not be adequately obtained through quantitative methods [45].

## Conclusion

Government efforts to promote early and regular ANC seeking behaviour and to increase accessibility to services through cost-reductions and subsidized prices all contribute to increasing health equity in Tanzania. This study has documented a variety of resource-related factors affecting the health seeking behaviour of pregnant women in rural Tanzania. Thus, accessibility to ANC services was hampered by direct and indirect costs, travel distances and waiting time. Strengthening of user-fee exemption practices and bringing services closer to the users, for example by promoting community-directed control of selected public health services, including IPTp, are measured urgently needed for increasing equity to health services in Tanzania.

## Competing interests

The authors declare that they have no competing interests.

## Authors' contributions

GM was a PhD student in this study and was therefore involved in all stages of the study from its conception through all the reports written about it. PB, JB, PM, ØEO, KSH and ICB supervised all stages of the study and commented on this manuscript. All authors read and approved the final manuscript.
